# Multi-organ damage induced by anabolic steroid supplements: a case report and literature review

**DOI:** 10.1186/1752-1947-2-340

**Published:** 2008-10-31

**Authors:** Ali A Samaha, Walid Nasser-Eddine, Elizabeth Shatila, John J Haddad, Jaafar Wazne, Ali H Eid

**Affiliations:** 1 Department of Internal Medicine, Makassed General Hospital, Beirut, Lebanon; 2 Department of Human Morphology, Faculty of Public Health, Lebanese University, Zahle, Lebanon; 3 Cellular and Molecular Signaling Research Group, Departments of Biology and Biomedical Sciences, Faculty of Arts and Sciences, Lebanese International University, Beirut, Lebanon; 4 Department of Nutrition and Dietetic, Faculty of Arts and Sciences, Lebanese International University, Beirut, Lebanon; 5 Clinical Laboratory, Faculty of Public Health, Lebanese University, Zahle, Lebanon; 6 Lebanese School of Social Formation: Community Health Program, Saint-Joseph University, Beirut, Lebanon; 7 Department of Biology, College of Science, United Arab Emirates University, Al-Ain, UAE

## Abstract

**Introduction:**

The use of anabolic supplements and other related drugs for body building and to enhance athletic performance is nowadays widespread and acutely pervasive all around the world. This alarming increase in the use of anabolic and amino acid supplements has been linked to a diverse array of pathologies. As previously reported, the abuse of androgenic steroids is not without severe physiological, psychiatric and physical costs. The case we report here describes multi-organ damage resulting from the abuse and uncontrolled use of anabolic steroid supplements, mainly testosterone.

**Case presentation:**

A 24-year-old white man presented with abdominal pain concomitant with nausea and vomiting. Laboratory analysis revealed hypercalcemia, elevated liver enzymes and high levels of amylase, lipase and creatine protein kinase.

**Conclusion:**

Amino acid as well as anabolic supplements may lead to abnormal functioning of many organs, which could be fatal in some instances. This mandates worldwide and concerted efforts to educate the public, especially the youth, about the dangers of these increasingly abused drugs.

## Introduction

Anabolic-androgenic steroids and amino acid supplements are abused by many individuals for a variety of reasons: to boost athletic performance, increase muscle mass or even to enhance their appearance [1]. The abuse of these drugs has been linked to many pathological conditions. For instance, it was recently shown that anabolic steroid abuse could lead to reduced fertility and increased cardiovascular diseases [2]. Severe depression was also reported in four men who had used anabolic-androgenic steroids for a long period of time [3]. Interestingly, many of the female steroid users developed a distorted image of their body, analogous to "reverse anorexia", wherein they viewed themselves as too small [4].

Although many of the undesirable effects of steroid abuse have been reported, little is known about the effect of anabolic supplements on the plasma levels of calcium. In addition, a possible relationship between hypercalcemia and the organ damage that could be induced by anabolic supplements, namely testosterone, has not been thoroughly discussed before.

## Case presentation

A 24-year-old white male smoker, previously healthy, presented to the emergency room (ER) of the Makassed General Hospital with abdominal pain of several days duration. The patient was 173 cm in height and weighed 85 kg. He described his pain as dull and continuous, worsening from time to time, mainly involving the epigastric area, radiating bilaterally to the back and associated with nausea and vomiting. Curiously, his pain was not provoked by food intake.

The patient had no history of alcohol intake. He exercised regularly and reported taking testosterone injections three times weekly for the past 2 months. He also reported the intake of diuretics and amino acid supplements. The patient reported no intake of other vitamin and mineral supplements.

Physical examination was normal except for diffuse abdominal tenderness elicited even with light palpation. Primary laboratory analysis showed leukocytosis with left shift, hypercalcemia, mildly elevated liver enzymes, elevated creatinine level, and a significant increase in the levels of amylase, lipase and creatine protein kinase (CPK) (Table 1). Negative ketones in the blood and normal urine analysis were read. An elevated serum calcium level of 13.8 mg/dl was measured, whereas the measured albumin level was near normal (3.3 g/dl). Taken together, these two values show a corrected calcium level of nearly 14.3 mg/dl. Further analysis showed a low parathyroid hormone (PTH) level indicating a suppressed parathyroid function as well as an increased level of 1,25 dihydroxy vitamin D.

**Table 1:** Admission laboratory results for the reported case

Bun (7.0–12.0 mg/dl)	Creatinine (0.2–1.2 mg/dl)	Amylase (30–110 U/liter)	Lipase (23–300 U/liter)	AST (0–50 U/liter)	ALT (0–50 U/liter)	GGT (1–60 U/liter)
52	5.2	717	8426	64	43	27

						
CPK (10–190 U/liter)	LDH (135–225 U/liter)	Na^+^ (130–145 mmol/liter)	K^+^ (3.5–5.4 mmol/liter)	Mg^2+^ (1.6–2.6 mmol/liter)	PO_4_^2-^ (2.5–4.5 mg/dl)	Ca^2+^ (8.5–10.5 mg/dl)

1253	264	141	4.65	0.8	3	13.8

The patient was admitted for management with a primary diagnosis of acute pancreatitis, acute renal failure and hypercalcemia.

After admission, the patient's vital signs were normal, with no fever or disturbances in pulse and respiratory rates. Electrocardiogram (ECG) assessment showed regular sinus rhythm, with no abnormalities. Chest X-ray revealed minimal bilateral basal pleural effusion. Abdominal ultrasound showed slightly enlarged liver, distended gall-bladder, dilated common bile duct (0.9 cm) with no evidence of calculi, as well as minimally enlarged spleen. The pancreas was surrounded by a minimal amount of fluid.

**Figure 1:**
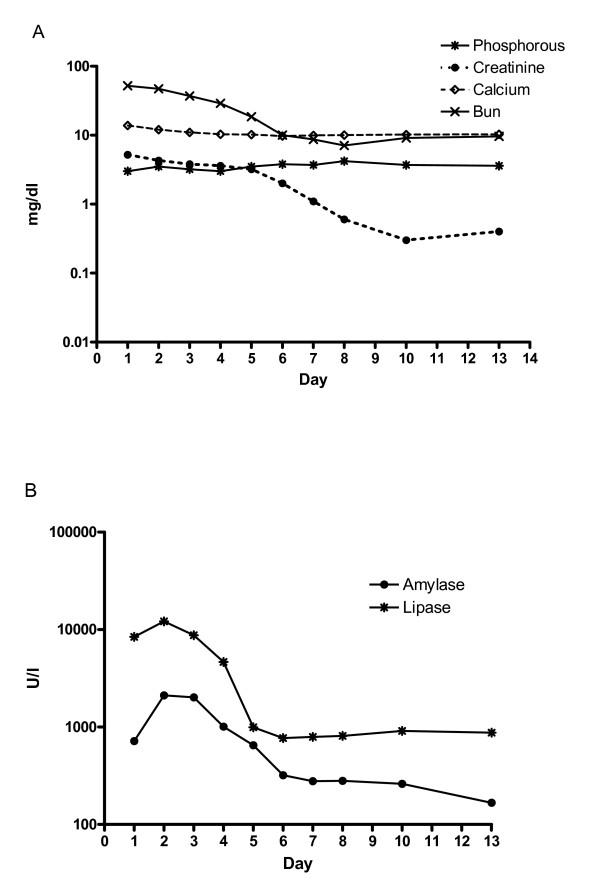
**Follow-up of different laboratory parameters during hospitalization of the reported case**. A) Levels of phosphorous, creatinine, calcium and blood urea nitrogen (BUN) (mg/dl). B) Levels of amylase and lipase (U/liter).

A computed tomography (CT) scan of the abdomen showed a swollen pancreas without any focal lesions or calcification. Management included aggressive fluid therapy, furosemide, proton pump inhibitors and symptomatic treatment. Due to pain severity, several injections of pethidine were required every day. Daily lab studies were taken for 10 days, after which the patient was discharged. The fluctuations of various laboratory measurements are shown in Figure 1.

## Discussion

The most likely cause of the patient's systemic and metabolic disturbances is hypercalcemia. As previously reported, constipation, anorexia, nausea and vomiting are often the prominent symptoms of hypercalcemia [5]. In addition, hypercalcemia has been associated with acute pancreatitis and peptic ulcer diseases that could be explained by the hypercalcemia-induced activation of trypsin and gastrin secretions, respectively [5]. Other symptoms of hypercalcemia include fatigue, musculoskeletal weakness and pain [6,7]. It has also been reported that acute renal failure and adrenal abnormalities are associated with hypercalcemia [6]. Therefore an overview of calcium homeostasis and a brief summary of the different kinds of anabolic and body building supplements could be helpful in understanding, interpreting and managing the reported case.

Calcium is critical for survival in higher organisms. Calcium and phosphorus are both absorbed into the body primarily in the duodenum and jejunum. In addition to the calcium ingested in diet, 600 to 700 mg is added from the intestinal secretions. Approximately 1600 to 1700 mg of calcium is present in the intestinal lumen, of which 700 mg is absorbed or reabsorbed into the bloodstream and is constantly exchanged with the calcium already present in extra and intracellular fluids of the body [7]. The entire extracellular pool of calcium turns over between 40 and 50 times daily. Renal reabsorption of calcium is very efficient under normal conditions and only between 100 and 200 mg of calcium appears in urine. In the case of hypercalcemia, urinary excretion may increase in a compensatory fashion and it may exceed 400 to 600 mg/day.

Regardless of race, all individuals have approximately the same calcium needs which may differ according to the stage of skeletal maturation, pregnancy, and/or lactation [5,7]. It is well accepted that the endocrine system is actively involved in calcium homeostasis. For example, the kidney produces and regulates the key metabolites of vitamin D by means of 25(OH) D_3_-1-hydroxylase and 25(OH) D_2_-24 hydroxylase activities [7]. Both hydroxylases are located in the mitochondria of the proximal convoluted tubules and both are cytochrome P_450_-containing enzymes. In their biochemical structures and properties, they are similar to steroid hydroxylases found in the adrenals, testes and ovaries [8]. Importantly, receptors for the 1,25(OH)_2_D_3 _form of vitamin D are expressed in cells of different organs such as the intestine, kidney and bones as well as pancreas, brain, pituitary gland, skin, and reproductive organs [9]. These receptors can also be activated by glucocorticoids, thyroxin, aldosterone and retinoic acid.

Testosterone is known to regulate many physiological processes including muscle protein metabolism, sexual and cognitive functions, secondary sexual characteristics, erythropoiesis, and bone metabolism [10]. It increases bone and skeletal muscle mass by enhancing the uptake of amino acids and increasing the serum level of insulin growth factor IGF I [11]. This non-genomic action of testosterone is mediated by secondary messengers such as calcium [11]. Calcium appears to be necessary not only for muscle contraction but also for activation of different energy pathways as well as cellular proliferation and maturation. Indeed, changes in fat-free mass, muscle volume, strength and power, as well as hemoglobin levels are positively correlated with testosterone levels while plasma HDL and fat mass are negatively correlated with testosterone levels [12]. Table 2 shows some of the most commonly abused anabolic androgenic steroids [10].

**Table 2:** Commonly abused anabolic steroids [2]

Intramuscular preparations	Oral preparations
Methenolone enanthate (Primobolan)	Fluoxymesterone (Halotestin)
Nandrolone decanoate (Deca dorabolin)	Mesterolone (Proviron)
Nandrolone phepropionate (Durabolin)	Oxandrolone (Anavar, Oxandrin)
Testosterone cypionate (Depotest)	Stanozolol (Winstrol)
Testosterone enanthate (Andro-estro)	
Testosterone propionate (Testex)	
Trenbolone acetate (Finajet)	

Several herbs are currently used to enhance physical performance. They can improve muscular strength, oxygen uptake, work capacity, fuel homeostasis, serum lactate level and heart rate. Some of these herbs are classified as adaptogens that assist in normalization of body system functions altered by stress rather than exerting a stimulatory effect. Others are used to improve performance, endurance, strength and to maintain health during intense periods of exercise [13]. Yet others are employed to build muscular mass and reduce body fat by means of their testosterone- and alpha adrenergic-like effects [13]. Table 3 summarizes the most common herbs used by body builders [13].

**Table 3:** Herbs commonly used in body building [14]

Herb	Reason for use
Arctic rose *(Rhodiola crenulata)*	Adaptogenic, enhances endurance and strength
Ashwagandha *(Withania somnifera)*	Adaptogenic, enhances endurance and strength
Asian ginseng *(Panax ginseng)*	Adaptogenic, enhances endurance and strength
Wild oats *(Avena sativa)*	Increases testosterone (anabolic effects)
Saw palmetto berries *(Serenoa repens)*	Testosterone-like effects
Chinese ephedra *(Ephedra sinica)*	Central nervous system stimulant, enhances endurance, strength and body fat loss
Yohimbe *(Pausinystalia yohimbe)*	Alpha adrenergic agonist, potentiates caffeine and ephedrine effects, increases male performance

The multi-organ damage in our patient could be explained by the hypercalcemia that had occurred most probably as a result of anabolic steroid injections. Anabolic steroids modulate steroid hydroxylase activity thereby precipitating hypercalcemia [12,13].

Besides hypercalcemia, acute pancreatitis could have resulted from the overuse of amino acid supplements. Notably, arginine was shown to be a potent secretagogue for anabolic hormones such as insulin and growth hormone in addition to inducing pancreatic acinar damage [14].

Acute renal failure can also be caused by the non-monitored use of diuretics in the presence of hypercalcemia, which may be due to elevated 25-OH-vitamin D [5]. Moreover, it has been reported that opiate analgesics are increasingly abused by anabolic steroid users as a means to reduce the pain induced by heavy training [10]. This could potentially explain the observation that our patient did not respond to the usual analgesics, forcing us to resort to pethidine.

## Conclusion

In our patient, we have mentioned some of the organic and systemic effects of anabolic supplement abuse without detailing their psychiatric effects that could be extremely variable and dangerous. Such effects include, but are not limited to, severe depression, bipolar disorders, panic attacks and others [10]. Moreover, the abuse of anabolic-androgenic steroids may be linked to the abuse of other substances. Indeed, one fourth of opiate users admitted to treatment centers acknowledged an earlier use of steroids [15]. Kanayama *et al. *also indicate that this link is often overlooked by most treatment centers [15]. Taken together, these data show the danger of the abuse of these anabolic steroids.

Disparity and lack of precise consistency of medical knowledge on these widely abused drugs together with their quick and uncontrolled spread among athletes and body builders mandate a worldwide collective endeavor to educate both the public and physicians about this issue. Specialized centers will be needed to provide and encourage medically-supervised withdrawal and give psychiatric support for abusers if this trend continues.

## Abbreviations

ALT: alanine aminotransferase; AST: aspartate aminotransferase; BUN: blood urea nitrogen; CPK: creatine protein kinase; CT: computed tomography; ECG: electrocardiogram; ER: emergency room; GGT: gamma glutamyl transferase; HDL: high density lipoprotein; LDH: lactate dehydrogenase; PTH: parathyroid hormone

## Competing interests

The authors declare that they have no competing interests.

## Authors' contributions

AAS, WNE, ES and JW dealt directly with the patient, ordered the laboratory exams and decided the treatment regimen. AAS, AHE and JJH analyzed and discussed the data as well as prepared the manuscript.

## Consent

Written consent was obtained from the patient for publication of this case report and any accompanying images. A copy of the written consent is available for review by the Editor-in-Chief of this journal.
